# Regulatory network rewiring for secondary metabolism in *Arabidopsis thaliana* under various conditions

**DOI:** 10.1186/1471-2229-14-180

**Published:** 2014-07-04

**Authors:** Qi Lv, Rong Cheng, Tieliu Shi

**Affiliations:** 1 Center for Bioinformatics and Computational Biology, Shanghai Key Laboratory of Regulatory Biology, the Institute of Biomedical Sciences and School of Life Science, East China Normal University, Shanghai 200241, China; 2 Shanghai Institute of Plant Physiology and Ecology, Shanghai Institutes for Biological Sciences, Chinese Academy of Sciences, Shanghai 200031, China

**Keywords:** Regulatory network, Rewiring, Secondary metabolism, Arabidopsis

## Abstract

**Background:**

Plant secondary metabolites are critical to various biological processes. However, the regulations of these metabolites are complex because of regulatory rewiring or crosstalk. To unveil how regulatory behaviors on secondary metabolism reshape biological processes, we constructed and analyzed a dynamic regulatory network of secondary metabolic pathways in Arabidopsis.

**Results:**

The dynamic regulatory network was constructed through integrating co-expressed gene pairs and regulatory interactions. Regulatory interactions were either predicted by conserved transcription factor binding sites (TFBSs) or proved by experiments. We found that integrating two data (co-expression and predicted regulatory interactions) enhanced the number of highly confident regulatory interactions by over 10% compared with using single data. The dynamic changes of regulatory network systematically manifested regulatory rewiring to explain the mechanism of regulation, such as in terpenoids metabolism, the regulatory crosstalk of RAV1 (AT1G13260) and ATHB1 (AT3G01470) on HMG1 (hydroxymethylglutaryl-CoA reductase, AT1G76490); and regulation of RAV1 on epoxysqualene biosynthesis and sterol biosynthesis. Besides, we investigated regulatory rewiring with expression, network topology and upstream signaling pathways. Regulatory rewiring was revealed by the variability of genes’ expression: pathway genes and transcription factors (TFs) were significantly differentially expressed under different conditions (such as terpenoids biosynthetic genes in tissue experiments and E2F/DP family members in genotype experiments). Both network topology and signaling pathways supported regulatory rewiring. For example, we discovered correlation among the numbers of pathway genes, TFs and network topology: one-gene pathways (such as δ-carotene biosynthesis) were regulated by a fewer TFs, and were not critical to metabolic network because of their low degrees in topology. Upstream signaling pathways of 50 TFs were identified to comprehend the underlying mechanism of TFs’ regulatory rewiring.

**Conclusion:**

Overall, this dynamic regulatory network largely improves the understanding of perplexed regulatory rewiring in secondary metabolism in Arabidopsis.

## Background

The researches on mechanism, function and evolution of plant secondary metabolism were traced back to about 60 years ago [1,2]. Secondary metabolic pathways lead to tens of thousands of products involved in various biological responding processes, under stimuli of specific external environmental stress elicitors as well as signal molecules of normal growth and development [3,4]. Secondary metabolisms of Arabidopsis are classified into five major groups (Additional file 1: Table S1): nitrogen-containing secondary compounds biosynthesis (NSCB), terpenoids biosynthesis (TB), sugar derivatives biosynthesis (SDB), phenylpropanoid derivatives biosynthesis (PDB) and flavonoids biosynthesis (FB) in AraCyc database [5]. Most nitrogen containing compounds, playing important roles in biological responses in plant defense and human nutrition [6,7], are regulated by MYB and bHLH members in transcription levels [8]. Sugar secondary derivatives, members of low molecular weight metabolites (mainly cyclic sugar alcohols), are associated with osmotic stress in higher plants [9]. Phenylpropanoids are constitutive compounds in certain tissues [10] or responding factors induced by stresses (such as UV, wounding, pathogen attack, low temperature and low iron level) [10-13]. And these metabolites are regulated by AtMYB21 (AT3G27810), AtMYB4 (AT4G38620), HY5 (AT5G11260), and CIP7 (AT4G27430) [8]. Flavonoids, a major metabolic branch derived from phenylalanine and malonyl coenzyme A, are regulated by MYB and bHLH family members [14,15]. Terpenoids, the largest secondary metabolic family irreplaceable in inner communication with: environment; plant growth; and development [16-18], are regulated by AP2/ERF, bHLH and MYB members [19]. The significant functions of these compounds make their regulators critical targets in genetic engineering applications for improving plant qualities, and for enzymes engineering TF is one kind of candidates [20]. However, metabolic engineering primarily concentrates on production of only one metabolite or a single metabolic gene and normally generates unexpected metabolic consequences–because metabolic pathways in plant intertwine one another to form a complex network; and perturbation of a single gene in the network usually have extensively effects on metabolic flux [21,22]. Therefore, regulatory mechanisms of biosynthetic genes are too complex to comprehensively reveal because of ‘biodiversity’ or ‘chemodiversity’, asking for system analysis rather than independent experiments.

**Figure 1:**
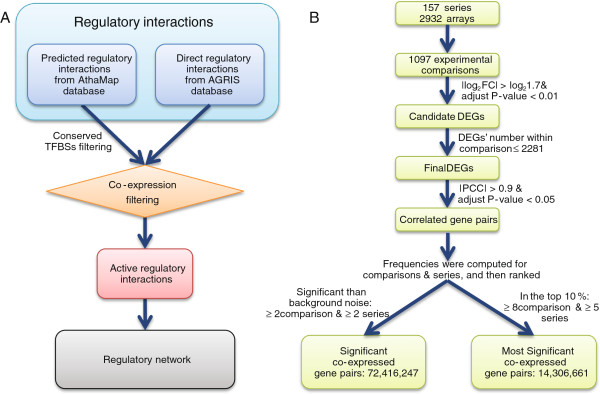
**Workflow of regulatory network construction. (A)** General procedure of generating regulatory network of secondary metabolism. **(B)** Strategy of processing microarrays. FC: fold change values; DEGs: differentially expressed genes; PCC: Pearson correlation coefficient; background noise: frequencies of randomly generated gene pairs.

The first sequenced flowering plant *Arabidopsis thaliana* is widely used as a model to systematically study gene function and physiology in plant science [23,24]. With high-throughput technologies such as microarray, Chip-chip etc., numerous data have been generated in this model plant, making it possible to explore biological mechanisms in plant developmental and environmental responses on genomic scale. Among these technologies, gene microarray aims to investigate expression of genes on a large scale in various treatments or developmental stages [25-28]. Many approaches used microarrays in systematic analysis of regulation over whole genome. For instance, Bayesian was applied to build dynamic regulatory network over time series microarrays, presuming causal relationships between TFs and target genes [29,30]. Other studies generated co-expression data from microarrays and then utilized function specific cis-elements (obtained from multiple sequence alignments on promoter regions of co-expressed genes) to reconstruct regulatory network, assuming that co-expressed genes are co-regulated by the same TFs [31]. Also, researchers used microarrays in expression quantitative trait locus (eQTLs) analysis to identify hot spot regions where regulatory genes locate. For example, researchers built genetic regulatory network in flowering and single gene mutants in Arabidopsis [32,33] and identified effects of TFs on multiple metabolic phenotypes [34]. However, these studies focusing on regulatory network–mainly stress (drought, cold, dehydration, etc.) or development (flowering, seed maturation, etc.) specific [35-38]–are limited in: types of experiments, sizes of networks, families of TFs and numbers of target genes. Besides, most available regulatory databases only addressed on their particular regulatory information (Additional file 2: Table S2). These limitations in regulatory network analysis and database specificity make it insufficient to systemically study regulatory mechanism–neglecting dynamic changes, biological responses and regulatory rewiring or crosstalk between regulations. However, systematic researches of transcriptional regulations on metabolic pathways are still fewer than function studies (only focusing on one or several TFs) [39,40].Therefore, we developed a method to construct a dynamic regulatory network significant in biological function by integrating regulatory interactions, large-scale microarray data and evolutionary conservation of TFBSs (Figure 1A). This dynamic network is efficient in systematically exploring regulatory rewiring (or crosstalk) on pathways to explain the mechanism of regulation. We investigated the regulatory rewiring with expression, network topology and upstream signaling pathways, which largely improves the understanding of perplexed regulatory rewiring mechanism in secondary metabolism.

## Results

### Dynamic regulatory network reconstruction with co-expression data and regulatory interactions

We reconstructed the regulatory network of secondary metabolism in *Arabidopsis thaliana* through combining co-expressed gene pairs with regulatory interactions (Figure 1A): either 422,967 predicted regulatory interactions from AthaMap [41], which were then filtered by conserved transcription factor binding sites (TFBSs); or 10,653 directly experiment-proved ones from AGRIS [42,43].

**Figure 2:**
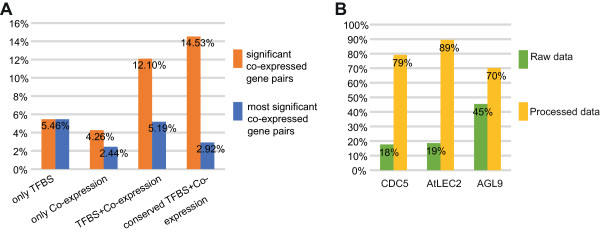
**Validation of filtering method in identifying high confident regulatory interactions. (A)** The percentages of experiment-confirmed regulatory interactions in regulatory interactions predicted by different data. To validate the filtering strategies of AthaMap data, the proportion of direct regulatory interactions (AGRIS) in the predicted ones (AthaMap) across different data filtering strategies were computed with the regulatory interactions of five TFs: FUS3 (AT3G26790), AtLEC2 (AT1G28300), AG (AT4G18960), AGL15 (AT5G13790) and HY5 (AT5G11260) present in both AGRIS and AthaMap. **(B)** Literature evidences about the function of TFs’ target pathways that were consistent with phenotypes of TFs’ mutant. The function of TFs’ target pathways were mined in literatures. The phenotypes of TFs’ mutant were obtained from AtPID (Arabidopsis thaliana Protein Interactome Database). The percentages of target pathways whose functions were consistent to TFs’ mutant phenotypes were computed.

We first used conserved TFBSs (see “Methods”) to increase the confidence of regulatory interactions predicted in AthaMap from the perspective of evolution. As expected, poplar (the closest species to Arabidopsis among four used organisms in the evolutionary tree) had more conserved TFBSs. In contrast, we did not find any conserved TFBSs among TB orthologous genes in chlamydomonas (the farthest species to Arabidopsis among four used organisms in the evolutionary tree)–possibly owing to large evolutionary distance between them. This verifies the rationale of our results in conserved TFBSs computing.Next, 72,416,247 significantly co-expressed gene pairs and 14,306,661 most significantly co-expressed gene pairs were obtained from microarray analysis (Figure 1B). Based on these co-expressed gene pairs, we identified a substantial amount of active regulatory interactions to construct regulatory network. 28% of regulatory interactions from AthaMap were maintained after being filtered with significantly co-expressed gene pairs. At the same time, among the regulatory interactions from AGRIS database, about 39% were significantly co-expressed and 6% were most significantly co-expressed–consistent with the fundamental assumption of regulatory interaction prediction: expression patterns of TFs and their target genes were similar.To validate our filtering strategies of AthaMap data, we compared the proportion of direct regulatory interactions (AGRIS) in the predicted ones (AthaMap) across different data filtering strategies (Figure 2A) with five TFs: FUS3 (AT3G26790), AtLEC2 (AT1G28300), AG (AT4G18960), AGL15 (AT5G13790) and HY5 (AT5G11260) in both AGRIS and AthaMap. After being filtered by only TFBSs alignments or significant co-expressed gene pairs, the percentages of direct regulatory interactions were 5.46% and 4.26% respectively. When being filtered by both TFBSs alignments and significantly co-expressed gene pairs, the fraction of direct regulatory interactions increased to 12.10%. After adding evolutionary conservation filtering, this percentage reached 14.53%. Therefore, our filtering methods were efficient in predicting highly confident regulatory interactions.

To verify that our processed regulatory interactions are more confident than raw regulatory interactions, we first compared the numbers of target pathways in these two datasets. We observed that the numbers of target pathways in our result were smaller than that in both raw dataset and random dataset (Additional file 3: Figure S1A). And the raw dataset was not significantly different from random dataset compared with our processed data (Additional file 3: Figure S1B). Furthermore, to validate the reliability of our proposed method in eliminating low confident data in raw dataset, we mined function of pathways (containing predicted target genes) from literatures and checked their consistency with TFs’ mutant-phenotypes in ATPID database [44]. Here we only chose TFs with simple mutant-phenotypes (Additional file 4: Table S3) to make the result more precise, ignoring complex mutant-phenotypes associated with multiple functions. The percentages of literature evidences in our processed data were higher than that in the raw dataset (Figure 2B): for each TF, functions of more than half target pathways in processed regulatory interactions were correlated with mutant-phenotype. In our results, CDC5 (AT1G09770, cell division cycle 5, a MYB family member) regulated 24 pathways, and 19 of them were correlated with embryo development in literatures–in accordance with embryonic defect, the phenotype of CDC5 mutant in ATPID. AGL9 (AT1G24260, agamous-like 9, a member of SEP3 family), whose mutant-phenotype was about flowers in ATPID, regulated 57 pathways in our result, and 40 of target pathways were associated with flowers in literatures. AtLEC2 mutant affected normal embryonic and cotyledonal development in ATPID, 17 of the 19 pathways which were predicted to be regulated by AtLEC2 were related to this TF’s mutant-phenotype. These results indicate that our method is efficient in identifying highly confident regulatory interactions from raw dataset.

### Regulatory rewiring under diverse conditions in TB

Based on the dynamic regulatory network, we analyzed regulatory rewiring to demonstrate the dynamic changes of regulation. In pathway level, according to the regulation of TFs on target genes, we classified regulations into three types: positive, negative or both positive and negative. Positive or negative regulation of a TF on a pathway signifies that the regulations on different pathway genes are constant and don’t change with experiments. Both positive and negative regulation, considered as inconstant and reciprocal regulation on different target enzymatic genes, maintains the balance of metabolic flux within pathways. For example, abscisic acid glucose ester biosynthesis contains only one reaction, and the reaction is catalyzed by abscisic acid glucosyltransferase that is encoded by more than 20 genes. We found both positive and negative regulations of RAV1 (AT1G13260, an AP2/B3 domain TF) on this pathway, which could keep abscisic acid glucosyltransferase steady.

Since TB is critical to plant, we took two examples in TB sub-network to illustrate regulatory rewiring under diverse experimental conditions. Generally, the occurrence of rewiring is caused by either regulatory interactions between TFs or regulatory alterations under different conditions.

One example is the rewiring between the regulatory crosstalk of RAV1 and ATHB1 (AT3G01470, a HD-ZIP family member) on HMG1 (AT1G76490, a hydroxymethylglutaryl-CoA reductase). RAV1 positively regulated HMG1 gene in independence (Additional file 3: Figure S2A) under many conditions: the grown stage of leaves; tocopherols mutant VTE1 (vitamin E deficient 1) gene; tocopherols mutant VTE2 (vitamin E deficient 2) gene; leaves responses to *Phytophthora infestans* and COP9 (constitutive photomorphogenic 9) signalosome mutants grown in dark, etc. However, the positive regulation role of RAV1 on HMG1 gene altered when ATHB1 promoted the expression of HMG1 gene (Additional file 3: Figure S2B) under a few conditions, like ABA1 (zeaxanthin epoxidase) gene mutant and hypoxia stress. We found that the distance between two TFs’ binding sites of HMG1 gene was within 200 bps, suggesting that the binding of ATHB1 on HMG1 gene affect normal binding of RAV1, and thereby change RAV1’s usual regulatory function. It implies that the interactions between these two TFs result in the alteration of RAV1 regulation on HMG1 gene.

The example of epoxysqualene biosynthesis also demonstrates the rewiring of regulatory crosstalk (Additional file 3: Figure S3). Epoxysqualene biosynthesis pathway is the upstream pathway, leading to sterol biosynthesis (a major class of triterpenoids) and other triterpenoids biosynthesis. The regulations of RAV1 on epoxysqualene biosynthesis and sterol biosynthesis pathways were the same in multiple experiments: Phytophthora infestans plants, water treatment on leaves for 24 hours, MgCl_2_ treatment on leaves for 12 hours, etc. But RAV1 regulated the two pathways differently under a few conditions (such as seedling and CSN3 gene mutant in dark): negatively regulated epoxysqualene biosynthesis but positively regulated sterol biosynthesis, because of the activation of some downstream biosynthetic genes regulated by RAV1 in sterol biosynthesis pathway. The change of RAV1’s regulation would affect normal metabolic distribution of sterol-related and the other triterpenoids, indicating the importance of regulatory rewiring in controlling metabolic flux.

### Variability of genes’ expressions revealing regulatory rewiring

**Figure 3:**
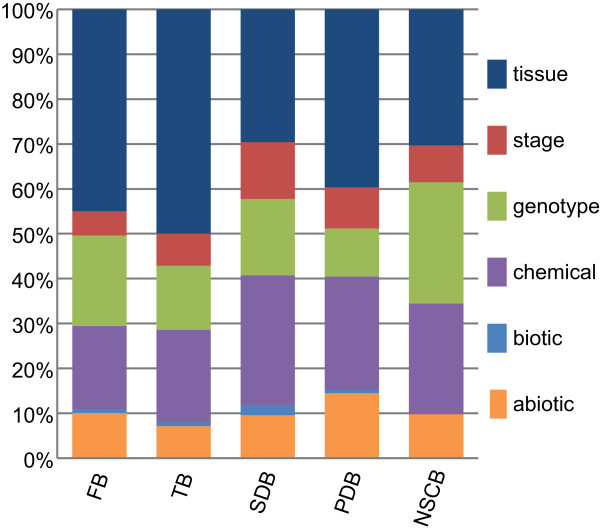
**The contrasts of six experiment categories for five secondary metabolic classes.** Within every metabolic class, DEGs’ numbers were counted for each experiment; and experiments were then ranked by DEGs’ numbers. Experiments above the top ten percent were used to compute the percentages of six experiment categories: tissue, stage, genotype, chemical, biotic and abiotic.

Significant variability in expressions of TF-encoding and pathway genes could further reveal regulatory rewiring by providing gene activities and functions specific to experimental conditions. We investigated the changes of gene activities and functions through differentially expressed genes (DEGs) (see “Methods”).Biosynthetic genes of FB, PDB and TB were significantly differentially expressed in tissue experiments (Figure 3), suggesting their dramatic changes in biological development of specific tissues. On the contrary, genes in NSCB were significantly differentially expressed in genotype experiments, indicating notable activities and biological function of nitrogen-containing compounds in plants of different genotypes.

The expressions of TF-encoding genes were also different under different experimental conditions. On one hand, genes–primary in WRKY(Zn), NAC, AP2/EREBP and MYB TF families–were widely differentially expressed (Additional file 3: Figure S4), indicating their global roles in regulations of downstream TFs or target enzymatic genes. In WRKY(Zn) family, WRKY18 (AT4G31800) and WRKY40 (AT1G80840) (which were induced by pathogen) [45], were greatly differentially expressed in genotype and chemical experiments; whereas WRKY6 (AT1G62300, associated with leaf senescence and defense) [46] was significantly differentially expressed in tissue and chemical treatments. Compared with WRKY(Zn) family, stress induced NAC family members (ANAC072, AT4G27410; ANAC019, AT1G52890; ANAC055, AT3G15500) [47] were differentially expressed in grown stages. On the other hand, TFs only differentially expressed in a few experiments included E2F/DP family (regulating core cell cycle) [48], C2H2(Zn) family (controlling flowering, germination and root development) [49-51] and ABI3/VP1 family (governing seed maturation) [52]. Those TFs were possibly more specific to particular conditions. For instance, a member of E2F/DP family (E2Ff, AT3G01330) was more specific to genotype experiments; whereas a member of C2H2(Zn) family (ID1, AT1G25250) was more typical to tissues.

To explore the relationship between TFs and pathway genes, we clustered them by expression profiles under different experiment categories and took TB as an example (Additional file 3: Figure S5). Firstly, we could not distinguish TFs and biosynthetic genes by two separated clusters. Secondly, one cluster was a union of similarly expressed genes, and contained both TFs and enzymatic genes, suggesting potential regulations between them in a cluster. Thirdly, clusters were different under five experiment categories, indicating variability of potential regulations depending on experimental conditions. This variability reflects biodiversity to a certain extent, which is in accordance with regulatory rewiring–emphasizing the reasons of biological complexity.

### Explanation of regulatory rewiring by network topology

**Figure 4:**
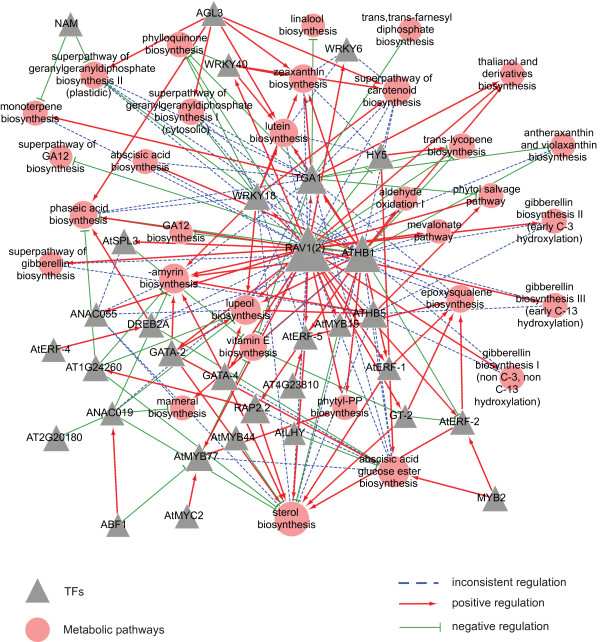
**Regulatory network of TB pathways.** Three types of regulation are presented in the network. Large size of TFs nodes in the central of the network are important to maintain regulatory network structure.

Network topological properties could efficiently explain regulatory rewiring based on network structure (Figure 4, Additional file 3: Figure S6-S9). We compared the general network properties of five secondary metabolic classes (Table 1). The numbers of TFs, genes and regulatory interactions in TB were the highest, indicating that the regulation of TB was the most complicated. In contrast, these topology properties in SDB were the lowest, revealing that the regulation of SDB was the simplest. The number of positive regulations was larger than that of negative regulations, and the number of inconsistent regulations was the least, which was discovered in network motifs as well. In addition, the network motifs (the basic component of the network) with high frequencies contained more positive regulations than negative regulations and inconsistent regulations, showing that most regulations were invariant (Additional file 3: Figure S10).

**Table 1:** Network properties of five secondary metabolic regulatory networks

	**No. of TFs**	**No. of genes**	**Average degree**	**No. of negative regulation**	**No. of positive regulation**	**No. of inconsistent regulation**
FB	25	57	4.15	47	101	22
NSCB	14	25	4.05	14	42	23
PDB	22	38	4.40	22	85	25
SDB	7	4	3.45	7	9	3
TB	32	72	4.12	58	118	38

FB, flavonoids biosynthesis; NSCB, nitrogen-containing secondary compounds biosynthesis; PDB, phenylpropanoid derivatives biosynthesis; SDB, sugar derivatives biosynthesis; TB, terpenoids biosynthesis; TFs, transcription factor; Average degree, the mean value of nodes’ degree to present network density.

Since high degree nodes are important in maintaining network structure whereas closeness gives a rough indication of how well a node connects the network, we used degree and closeness to explore the functions of pathways and TFs. We found that the nodes with high degree were also high in closeness (Additional file 3: Figure S11A). Interestingly, almost all nodes with both high degrees and closeness were TFs, while pathways always had low degree and closeness, indicating that TFs play a key role in maintaining the structure of network.

It is intriguing which factor affects the numbers of TFs in network. We discovered that the number of TFs was correlated with the number of genes in this pathway (Additional file 3: Figure S11B): the fewer genes in a pathway, the fewer TFs regulating this pathway, suggesting simpler regulation of this pathway. Besides, similar distributions of TFs’ numbers (Additional file 3: Figure S12) and pathway genes’ numbers (Additional file 3: Figure S13) in terpenoids metabolism also demonstrate the relevance; and the two numbers were all correlated with topological property. For example, one gene pathways (such as β-caryophtllene biosynthesis, δ-carotene biosynthesis and arabidiol biosynthesis) were regulated by a small number of TFs, indicating simple regulations; and these pathways were not critical to metabolic network, because of their low degrees in topology (shown as small nodes with light color in Additional file 3: Figure S12-13). However, pathways with more genes (like genranylgeranyl diphosphate, nonaprenyl diphosphate biosynthesis and epoxysqualene biosynthesis) were regulated by more than 20 TFs and were hub pathways in terpeniod metabolism (shown as large nodes with deep color in Additional file 3: Figure S12-13). The regulations of these pathways were complicated so that perplexing regulatory rewiring often occurred and led metabolic fluxes flowing into disparate downstream pathways. In other words, regulatory rewiring happened with changing conditions, and in turn affected metabolic flux within inner pathway or between different downstream pathways. These examples demonstrate that both simple and complex regulations can adapt to function of metabolic pathways, either specific or extensive.

### Contribution of TFs’ upstream signaling pathways to regulatory rewiring

Since signaling pathways regulate the activities of TFs, they contribute to TF’s regulatory rewiring. To define upstream signaling influences on TFs, we computed significances of expression correlations between plant signaling pathway genes and TFs (see “Methods”, Additional file 3: Figure S14). Plant signaling molecules are mainly metabolites, such as jasmonate (fatty acid derivatives biosynthesis), ethylene (methionine biosynthesis), brassinosteroid (terpenoids biosynthesis) and cytokinin (terpenes biosynthesis). We found that TFs involved in certain signaling pathways were truly significantly co-expressed with related signaling pathway genes. For example, AtMYC2 (AT1G32640, a MYC-related transcriptional activator), in the downstream of jasmonate signaling pathway, was significantly correlated with genes in this pathway; ARR1 (AT3G16857, response regulator 1) and ARR2 (AT4G16110, response regulator 1), activated by cytokinin indirectly, were significantly co-expressed with cytokinin signaling pathway genes. These results indicate the efficiency of predicting TFs’ upstream signaling pathways. Totally we found that 50 TFs were significantly correlated with 3 signaling pathways (Additional file 5: Table S4). Among the three signaling pathways, jasmonate and cytokinin signaling pathways were correlated with 45 TFs and 31 TFs respectively while ethylene signaling pathway was only correlated with 12 TFs: suggesting global regulatory function of jasmonate and cytokinin compared to ethylene. In addition, 11 TFs were correlated with three signaling pathways, and 16 TFs were associated with both jasmonate and cytokinin signaling pathways–indicating complicated regulations of signaling pathways on these TFs. Besides, the rest 23 TFs were correlated with only one signaling pathway, implying specific regulations of signaling pathways on these TFs. For example, RAV1 was significantly correlated with cytokinin signaling pathway, demonstrating potential regulation of cytokinin on RAV1. Here, the identification and summary of potential signaling pathways for TFs could largely improve the understanding of TFs’ regulatory rewiring.

## Discussion

Here we presented a method to construct dynamic regulatory network of secondary metabolic pathways. Based on the dynamic regulatory network, we systematically explored complicated regulatory rewiring or crosstalk occurring under distinct experimental conditions, and investigated the relationships between regulatory rewiring and expression, network topology and signaling pathway to unveil the complex regulatory mechanism. The major assumption of our method is that active regulatory interactions are co-expressed which was also applied in previous studies [53-55]. The activation and inhibition effects can be distinguished by correlation coefficient of TFs and their target genes. Our method of integrating multiple data is efficient in identifying high confident regulatory interactions. As described in result, regulatory interactions determined by three types of data were more reliable than those predicted by single or two type(s) of data. However, most significant co-expression relations are not efficient in prediction because strict criteria of co-expression would filter out gene pairs only co-expressed in a few experiments (this is also why we manipulated arrays within experimental comparisons but not whole arrays).

Based on regulatory alterations at pathway level (rather than genes), we mined regulatory rewiring to comprehensively understand the regulation mechanism of biological metabolic fluxes. For instance, co-regulation of two pathways may attribute to multi-functions of pathway genes shared by these two pathways. Besides, some TFs were considered as dominant regulators because of their frequently changed activities: such as RAV1, which widely regulates growth and developmental genes in Arabidopsis [56]. Furthermore, our result explained the mechanism of TFs regulation on metabolic pathways. For example, flavonoid biosynthesis is influenced by AtLEC2, HY5 and AGL15 [57-59]. Based on our result we discovered these TFs’ potential target genes, encoding flavanone 3β-hydroxylase, acetyl-CoA synthetase, 4-coumarate-CoA ligase and naringenin chalcone synthase. And the target genes encoding flavanone 3β-hydroxylase and 4-coumarate-CoA ligase could contribute to regulatory rewiring of HY5, while genes encoding 4-coumarate-CoA ligase and acetyl-CoA synthetase might be the reason of AGL15’s regulatory rewiring.

**Figure 5:**
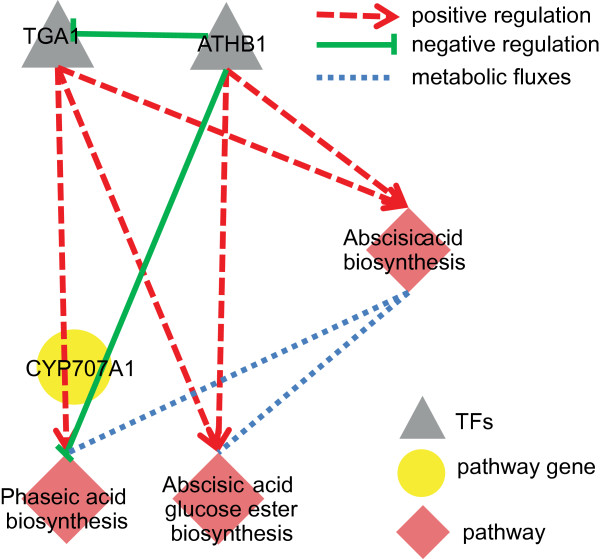
**Regulatory interactions between TGA and ATHB1 in abscisic acid biosynthesis.** The three abscisic acid metabolic pathways are regulated differently by both TGA1 and ATHB1, The two TFs reciprocally regulate CYP707A1 (AT4G19230, an abscisic acid 8’-hydroxylase) gene in phaseic acid biosynthesis pathway. The regulation of ATHB1 on three pathways cooperates with its negative regulation on TGA1’s expression.

Moreover, our work benefits plant metabolic engineering. A persuasive example is the regulatory crosstalk in abscisic acid metabolism (Figure 5). Abscisic acid biosynthesis is followed by two downstream pathways, abscisic acid glucose ester biosynthesis and phaseic acid biosynthesis. The three pathways are regulated differently by both TGA1(AT5G65210, a bZIP family member) and ATHB1: TGA1 positively regulates three pathways, whereas ATHB1 negatively regulates phaseic acid biosynthesis and positively regulates the other pathways. ATHB1 and TGA1 reciprocally regulate CYP707A1 (AT4G19230, an abscisic acid 8’-hydroxylase) gene in phaseic acid biosynthesis pathway; and the distance between two TFs’ binding sites on CYP707A1 promoter is within 200 bps–indicating spatial physical effects of the two TFs on their normal binding processes [55,60]. Besides, regulation of ATHB1 on three pathways cooperates with its negative regulation on TGA1’s expression, suggesting ATHB1 should be a key factor in abscisic acid metabolic regulation. Practically, we could overexpress ATHB1 to increase the yield of abscisic acid glucose ester (playing a potential physiological role under water stress) [61] and inhibit phaseic acid metabolic branch at the same time. In conclusion, this example of pathway crosstalk provides a reference to biologists on how to control metabolic products to improve desired plant traits in metabolic engineering.

We also notice that some TFs are not included in our result. This limitation attributes to restricted data sources and incomplete knowledge of regulation mechanism. Firstly, regulatory interactions collected from two databases are incomplete. For example, both AthaMap and AGRIS databses didn’t collect some well-known TFs (such as MYB28, MYB29, MYB34, MYB90, MYB12, MYB11, MYB4, MYB58, MYB63, TTG1, MYB75, etc.) and complete regulatory interactions (such as TT8 and TTG2, only have one target gene respectively in AGRIS database). Besides, the number of available microarrays is limited and insufficient to cover various experiments where TFs function–so that some TFs were filtered in microarrays analysis. Secondly, even if data collected in the databases were complete, not all functional TFs can meet our basic assumption and show significance in co-expression, because present knowledge of regulation is unable to definitely identify how well TFs’ expressions reflect their function switches. Therefore, the incompleteness of both data-collection and regulation mechanisms impacts the results, which is a common issue in systematic analysis.

## Conclusion

This systematic network-based bioinformatics approach largely improves the understanding of perplexed regulatory rewiring mechanism in secondary metabolism and provides useful references for biological experiments, especially metabolic engineering. The approach of reconstructing regulatory network and analyzing regulatory rewiring can be applied to comprehend the whole metabolism in Arabidopsis.

## Methods

### Data preparation

157 Affymetrix Arabidopsis ATH1 Genome Array platform (GPL198) series of microarrays with complete annotation and more than two duplications (Additional file 6: Table S5) were downloaded from Gene Expression Omnibus (GEO) [62]. Based on annotations, the experimental conditions could be clustered into six categories: biotic stresses, abiotic stresses, genotype experimental comparisons, chemical treatments, tissue experimental comparisons and grown stages. 1,097 experimental comparisons between two treatments within series (batches of experiments) were made for further analysis (Additional file 7: Table S6). Metabolic genes, enzymes and pathways were collected from AraCyc, a biochemical pathway database for Arabidopsis [5] (Additional file 8: Table S7). TFBSs of TFs and 422,967 regulatory interactions predicted by TFBSs alignments were collected from AthaMap [41,63]. 10,653 direct regulatory interactions between TFs and target genes confirmed by experiments from Arabidopsis gene regulatory information server (AGRIS), were used as both positive data and supplement of predicted regulatory interactions. 5 signaling pathways involving 93 genes in Arabidopsis were collected from Database of Cell Signaling (http://stke.sciencemag.org/cgi/collection/pw_plants) (Additional file 9: Table S8).

### Microarray data processing

Raw microarrays were preprocessed with RMA function in Bioconductor. Then Limma package in Bioconductor was applied to compute fold change values and P-values of all genes in each experimental comparison. We ranked genes in a descendent order by their absolute fold change values and then selected the top ten percent genes (absolute values of fold change larger than 1.7) with adjusted P-value less than 0.01 as candidate differentially expressed genes (DEGs). Raw P-values were adjusted by Benjamini & Hochberg method in p.adjust function. We kept the number of final DEGs no more than 2,281 (ten percent of all genes designed on GPL198 platform) to make DEGs more meaningful in both technological and biological sense, in consideration of microarray assumption that only a small number of genes are differentially expressed under different conditions.

### Computation of significantly and most significantly co-expressed gene pairs

For each experimental comparison, we calculated Pearson Correlation Coefficient (PCC) for each differentially expressed gene pairs using cor function in R. Then we tested the correlation coefficient by cor.test function in R and maintained gene pairs with absolute value of correlation coefficient bigger than 0.9 and adjusted P-value less than 0.05 as correlated gene pairs. Raw P-values were adjusted by Benjamini & Hochberg method in p.adjust function. Considering that the correlated gene pairs appearing only in one experimental comparison or one series might occur by chance, we further measured the statistical significance of correlated gene pairs as the following procedure similar with previous method [64]. We randomly generated gene pairs, keeping the same degree distribution and number of correlated gene pairs within one experimental comparison or series; and then calculated average frequencies of randomly generated gene pairs for experimental comparisons and series respectively. For each experimental comparison or series, DEGs were designated with non-duplicate genes randomly selected; and randomly co-expressed genes pairs were generated by replacing DEGs of original co-expressed gene pairs by the DEGs’ designated random genes. The frequencies of these random gene pairs were then counted, with mean value being defined as one random frequency. After repeating this procedure for 100 times, the mean values of random frequencies in experimental comparisons (1.38) and series (1.63) were obtained respectively, with standard deviation less than 0.01. Therefore, correlated gene pairs present in 2 or more experimental comparisons and series were defined as significantly co-expressed gene pairs. Most significantly co-expressed gene pairs were defined as the top ten percent of all correlated gene pairs, the frequencies of which were sorted in a descendent order.

### Regulatory network reconstruction and analysis

Co-expressed gene pairs obtained above were used to filter regulatory interaction pairs from both AthaMap and AGRIS, resulting in two sets of co-expressed regulatory interaction pairs. The set of co-expressed regulatory interaction pairs from AthaMap was further filtered by conserved TFBSs. The conserved TFBSs were those detected by sequence alignments in the upstream 3000 bps of transcription starting sites of four sequenced species (Populus trichocarpa, Sorghum bicolor, Brachypodium distachyon and Chlamydomonas reinhardtii) whose genome data were available from Phytozome database (http://www.phytozome.net/). If TFBSs of TFs with co-expressed regulatory interactions were conserved in these four organisms, related co-expressed regulatory interaction pairs were maintained. As the procedure described in Figure 1, we obtained complete regulatory interactions used for building the regulatory network of secondary metabolism in Arabidopsis. The final network of metabolic pathways was constructed by mapping enzymatic genes to pathways (Additional file 10: Table S9 and Additional file 11: Table S10). Then the topology properties of this network were computed by functions in igraph package in R, and network motif analysis was carried out by FANMOD. Frequent regulatory patterns were defined as the regulatory interaction pairs significantly simultaneously occurred.

The significance of co-expression relationship between signaling pathways and TFs were tested by fisher.test function in R. Finally, the signaling pathways with adjusted P-value less than 0.05 were regarded as significantly correlated with TFs. Raw P-values were adjusted by Benjamini & Hochberg method in p.adjust function.

## Abbreviations

DEGs: Differentially expressed genes; FB: Flavonoids biosynthesis; NSCB: Nitrogen-containing secondary compounds biosynthesis; PDB: Phenylpropanoid derivatives biosynthesis; SDB: Sugar derivatives biosynthesis; TB: Terpenoids biosynthesis; TFs: Transcription factors; TFBSs: Transcription factor binding sites.

## Competing interests

The authors declare that there are no competing interests.

## Authors’ contributions

QL collected all the datasets, analyzed raw data, wrote the draft and participated in study design. RC prepared figures and tables, participated in data collection and analysis, and helped to revise draft. TS designed the study and helped to draft and finalized the manuscript. All authors read and approved the final manuscript.

## Supplementary Material

Additional file 1: Table S1 Classification of secondary metabolism. Click here for file

Additional file 2: Table S2 List of Arabidopsis regulatory databases. Click here for file

Additional file 3 **Figure S1.** Significant performance of processed regulatory interactions. **Figure S2.** The regulatory influence of RAV1 on HMG1. **Figure S3.** Regulatory rewirings of RAV1 on epoxysqualene biosynthesis and sterol biosynthesis. **Figure S4.** The contrasts of six experiment categories for differentially expressed TFs. **Figure S5.** The expression profiles of TF-encoding genes and TB genes under five categories of experimental conditions. **Figure S6.** Regulatory network of SDB metabolic pathways. **Figure S7.** Regulatory network of PDB metabolic pathways. **Figure S8.** Regulatory network of FB metabolic pathways. **Figure S9.** Regulatory network of NSCB metabolic pathways. **Figure S10.** Motifs with highest frequencies in secondary metabolic regulatory network. **Figure S11.** Relationship of TF-encoding genes and pathways in the network. **Figure S12.** Distribution of TFs’ numbers in TB metabolic pathway network. **Figure S13.** Distribution of pathway genes’ numbers in TB metabolic pathway network. **Figure S14.** Significance of co-expression relationship between signal pathways and TFs. Click here for file

Additional file 4: Table S3 List of predicted regulatory interactions, TF mutant phenotypes in ATPID and pathways’ function mined from literatures. Click here for file

Additional file 5: Table S4 Significantly correlated TFs and signaling pathways. Click here for file

Additional file 6: Table S5 Information of applied microarray data. Click here for file

Additional file 7: Table S6 Information of experimental comparisons in microarray analysis. Click here for file

Additional file 8: Table S7 Information of AraCyc pathways. Click here for file

Additional file 9: Table S8 5 signaling pathways of Arabidopsis in Database of Cell Signaling. Click here for file

Additional file 10: Table S9 Regulatory network of secondary metabolic pathways (filtered by significantly co-expressed gene pairs). Click here for file

Additional file 11: Table S10 Regulatory network of secondary metabolic pathways (filtered by most significantly co-expressed gene pairs). Click here for file
