# Supplementary material for: Regulatory network rewiring for secondary metabolism in Arabidopsis thaliana under various conditions
Source: BMC Plant Biol. 2014 Jul 4;14:180. doi: 10.1186/1471-2229-14-180 (PMC4105546; doi:10.1186/1471-2229-14-180)
Supplement: Additional file 3 — Figure S1. Significant performance of processed regulatory interactions. Figure S2. The regulatory influence of RAV1 on HMG1. Figure S3. Regulatory rewirings of RAV1 on epoxysqualene biosynthesis and sterol biosynthesis. Figure S4. The contrasts of six experiment categories for differentially expressed TFs. Figure S5. The expression profiles of TF-encoding genes and TB genes under five categories of experimental conditions. Figure S6. Regulatory network of SDB metabolic pathways. Figure S7. Regulatory network of PDB metabolic pathways. Figure S8. Regulatory network of FB metabolic pathways. Figure S9. Regulatory network of NSCB metabolic pathways. Figure S10. Motifs with highest frequencies in secondary metabolic regulatory network. Figure S11. Relationship of TF-encoding genes and pathways in the network. Figure S12. Distribution of TFs’ numbers in TB metabolic pathway network. Figure S13. Distribution of pathway genes’ numbers in TB metabolic pathway network. Figure S14. Significance of co-expression relationship between signal pathways and TFs. [file 1471-2229-14-180-S3.pdf]

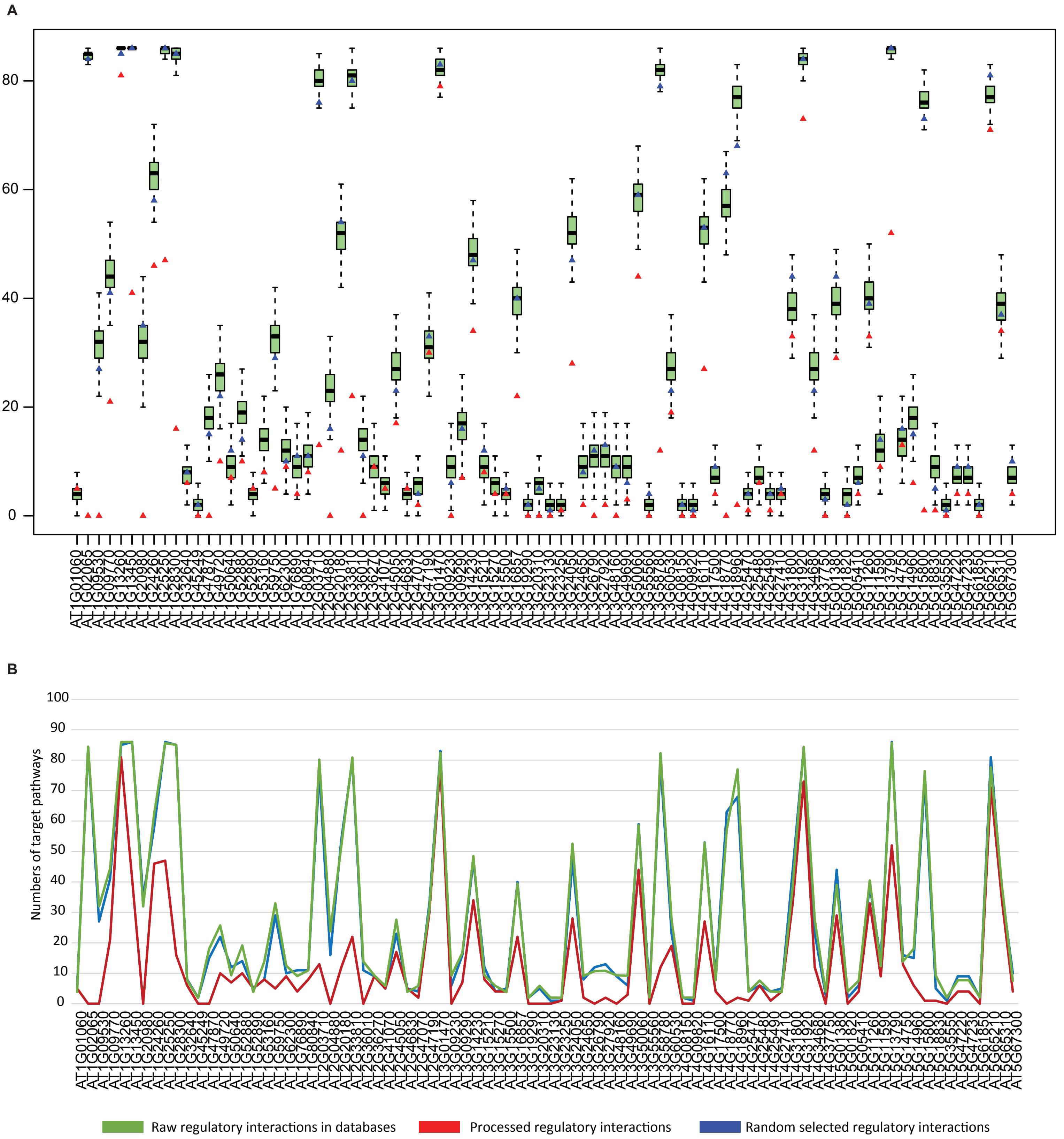

Supplementary Figure S1: Significant performance of processed regulatory interactions. (A) Numbers of target pathways in processed regulatory interactions and raw dataset compared with random generated dataset. **(B)** Numbers of target pathways in processed regulatory interactions and raw dataset compared with average number of random target pathways. For each pathway, random genes were generated with the same pathway gene number. And random genes' regulatory interactions were used to generate random target pathways of TFs. This procedure was repeated 1,000 times to draw the boxplot and to compute the average number of random target pathways.

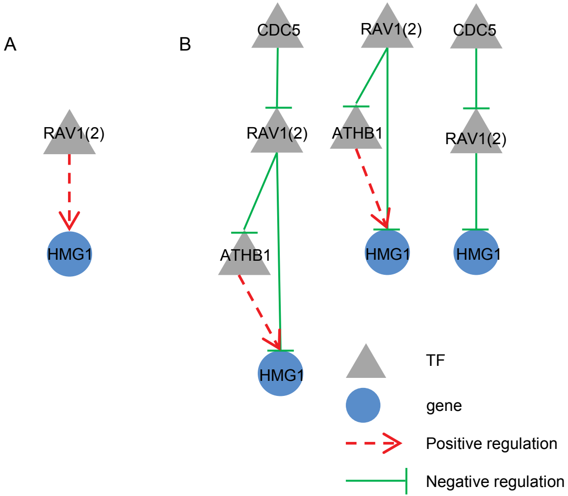

Supplementary Figure S2: The regulatoy influence of RAV1 on HMG1. (A) RAV1 positively regulates HMG1 independently. **(B)** RAV1 negatively regulates HMG1.

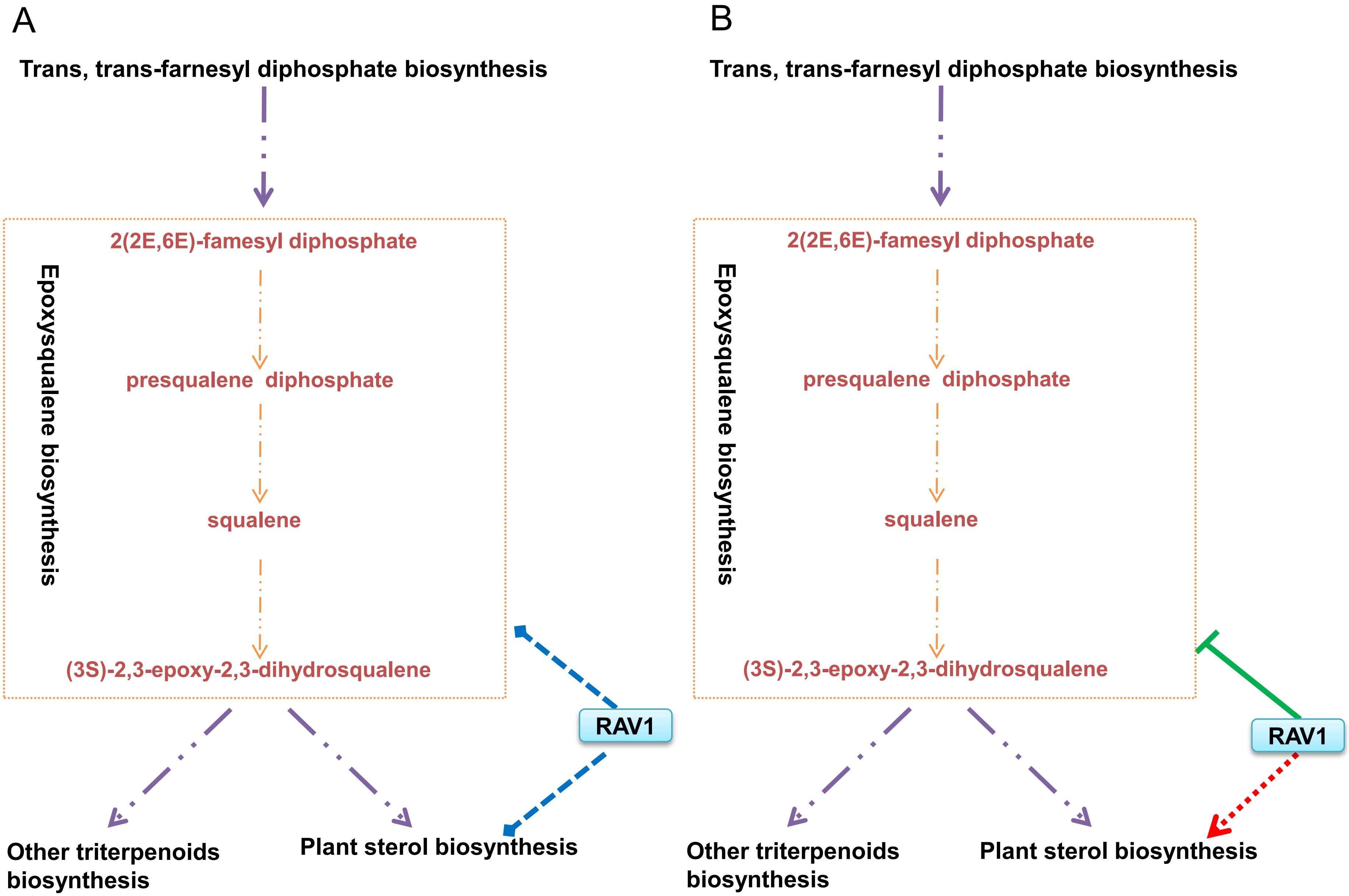

Supplementary Figure S3: Regulatory rewirings of RAV1 on epoxysqualene biosynthesis and sterol biosynthesis. **(A)** RAV1 regulated epoxysqualene biosynthesis and plant sterol biosynthesis in the same way. **(B)** RAV1 negative regulated epoxysqualene biosynthesis and positive regulated plant sterol biosynthesis.

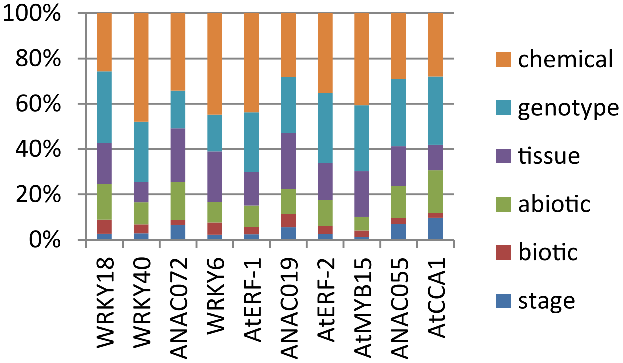

Supplementary Figure S4: The contrasts of six experiment categories for differentially expressed TFs. (TFs were ranked by numbers of experiments when they were differentially expressed; and the top ten TFs were used to compute the percentages of six experiment categories: tissue, stage, genotype, chemical, biotic and abiotic.)

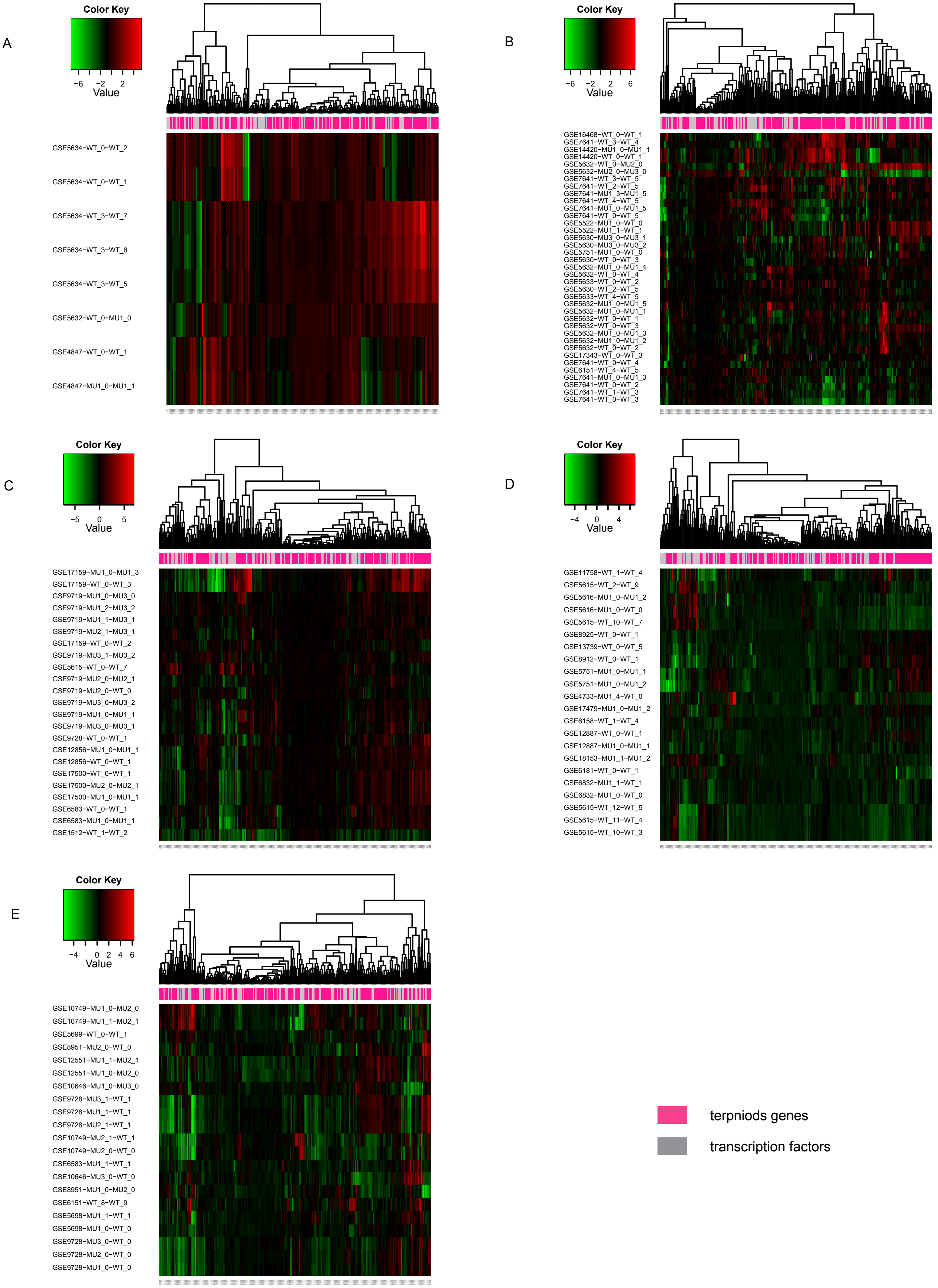

Supplementary Figure S5: The expression profiles of TF-encoding genes and TB genes under five categories of experimental conditions: **(A)** Grown and developmental stages; **(B)** Tissues; **(C)** Abiotic stresses; **(D)** Chemical treatments; **(E)** Genotypes.

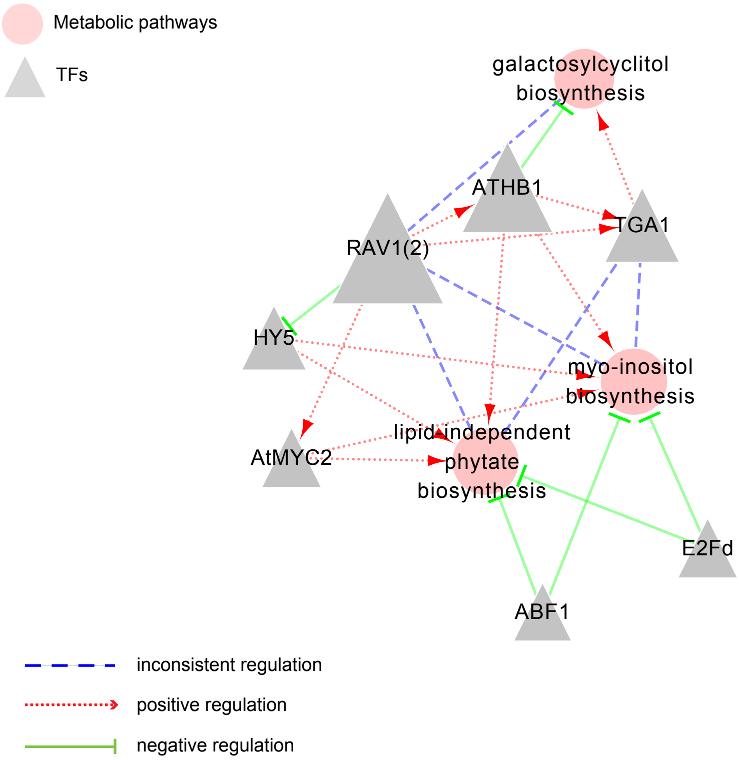

Supplementary Figure S6: Regulatory network of SDB metabolic pathways.

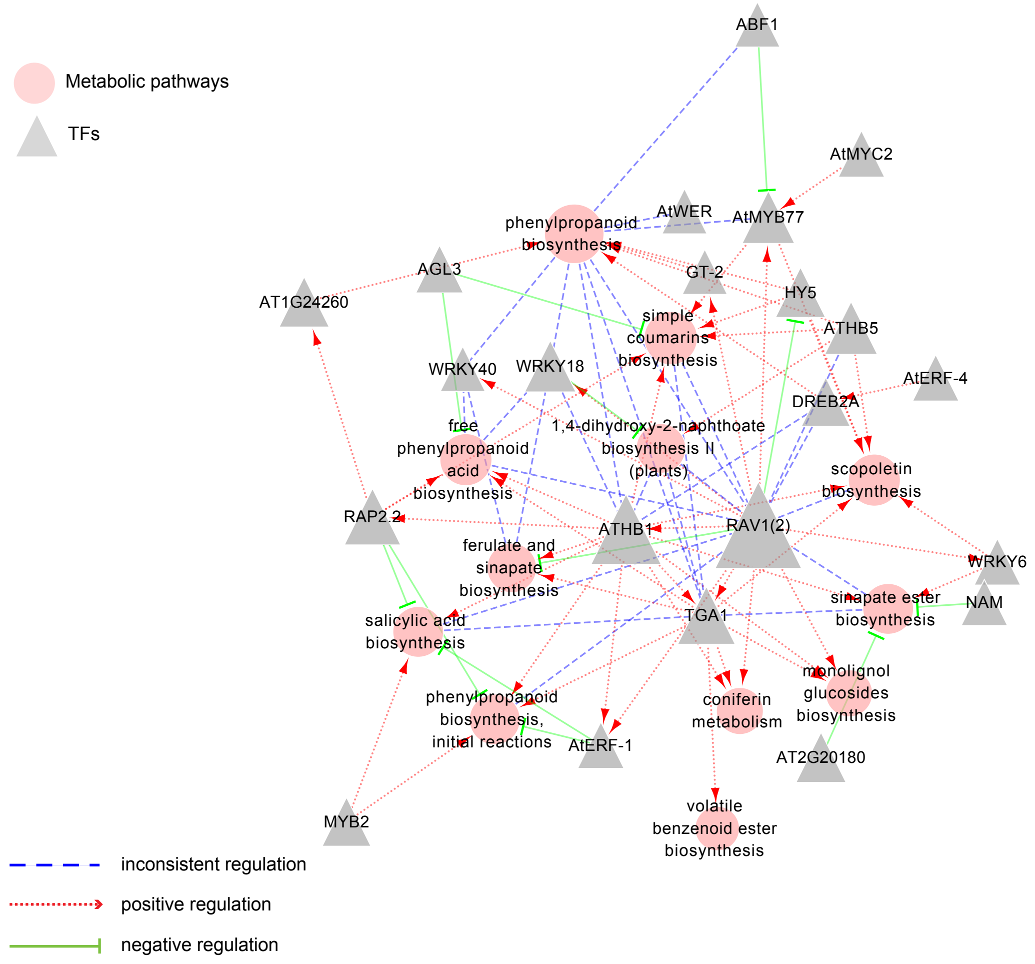

Supplementary Figure S7: Regulatory network of PDB metabolic pathways.

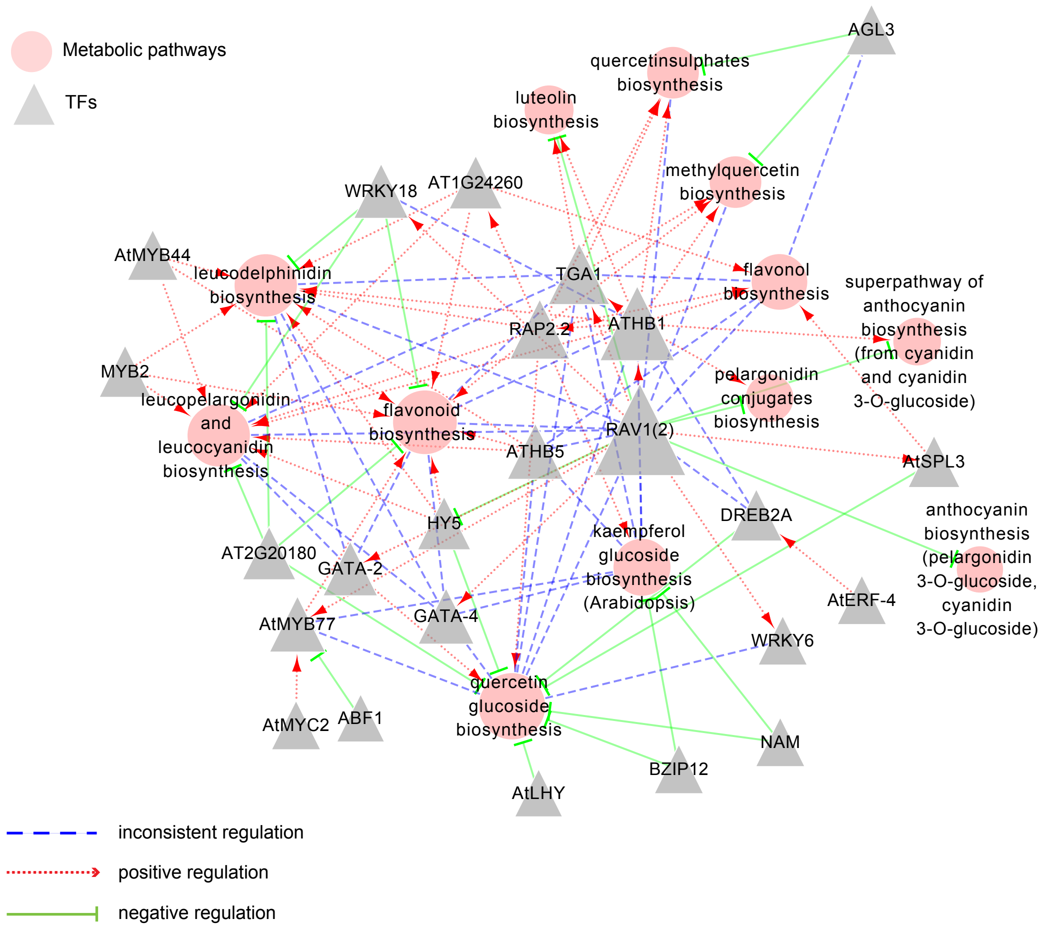

Supplementary Figure S8: Regulatory network of FB metabolic pathways.

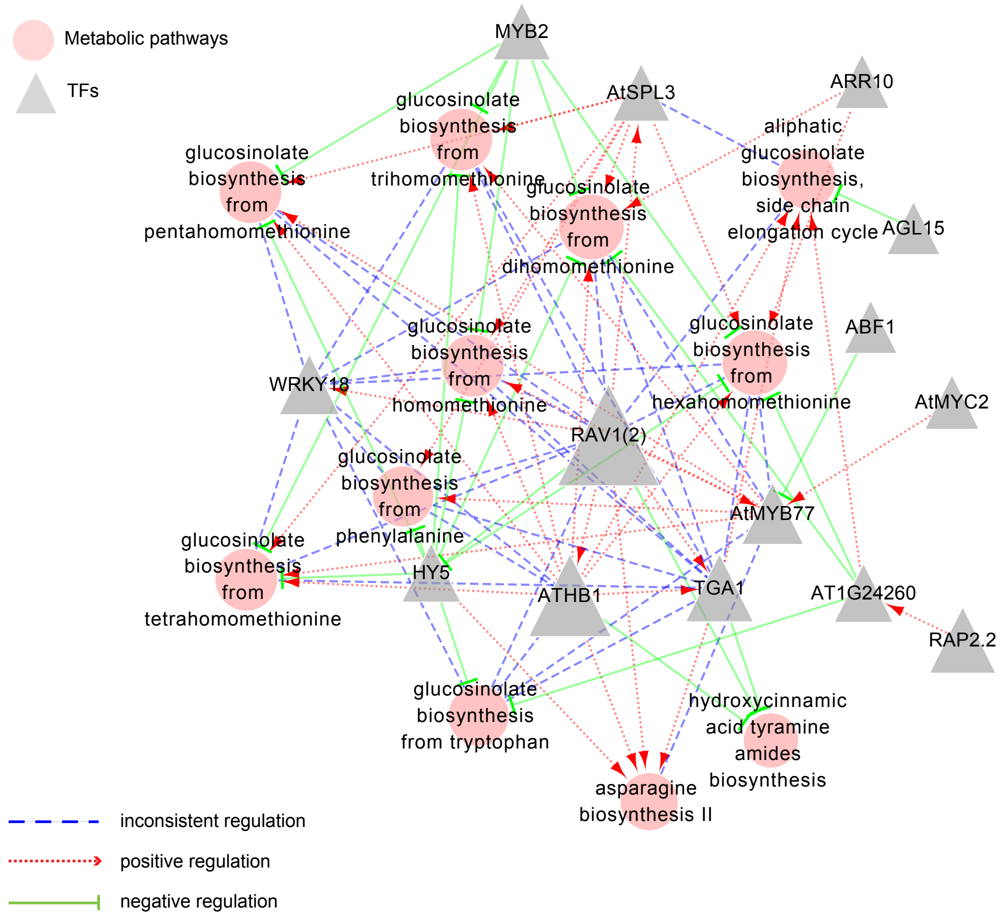

Supplementary Figure S9: Regulatory network of NSCB metabolic pathways.

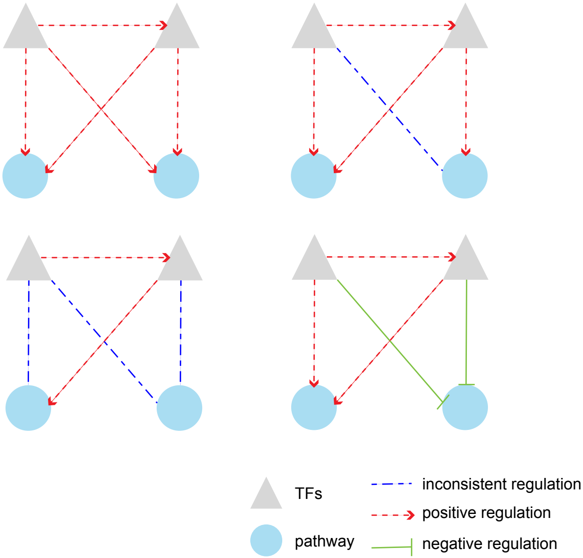

Supplementary Figure S10: Motifs with highest frequencies in secondary metabolic regulatory network.

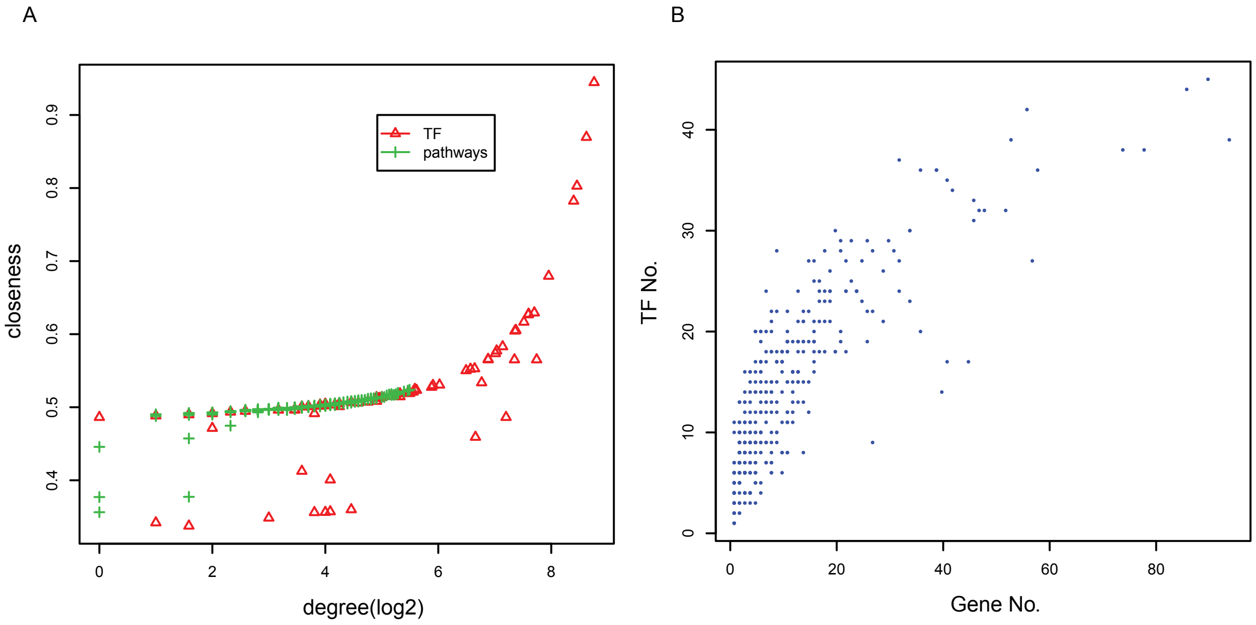

Supplementary Figure S11: Relationship of TF-encoding genes and pathways in the network. (A) The relationship of closeness and degree in both pathways and transcription factors. **(B)** The influence of pathway genes' number on TFs' number per pathway. (Each node represents a pathway.)

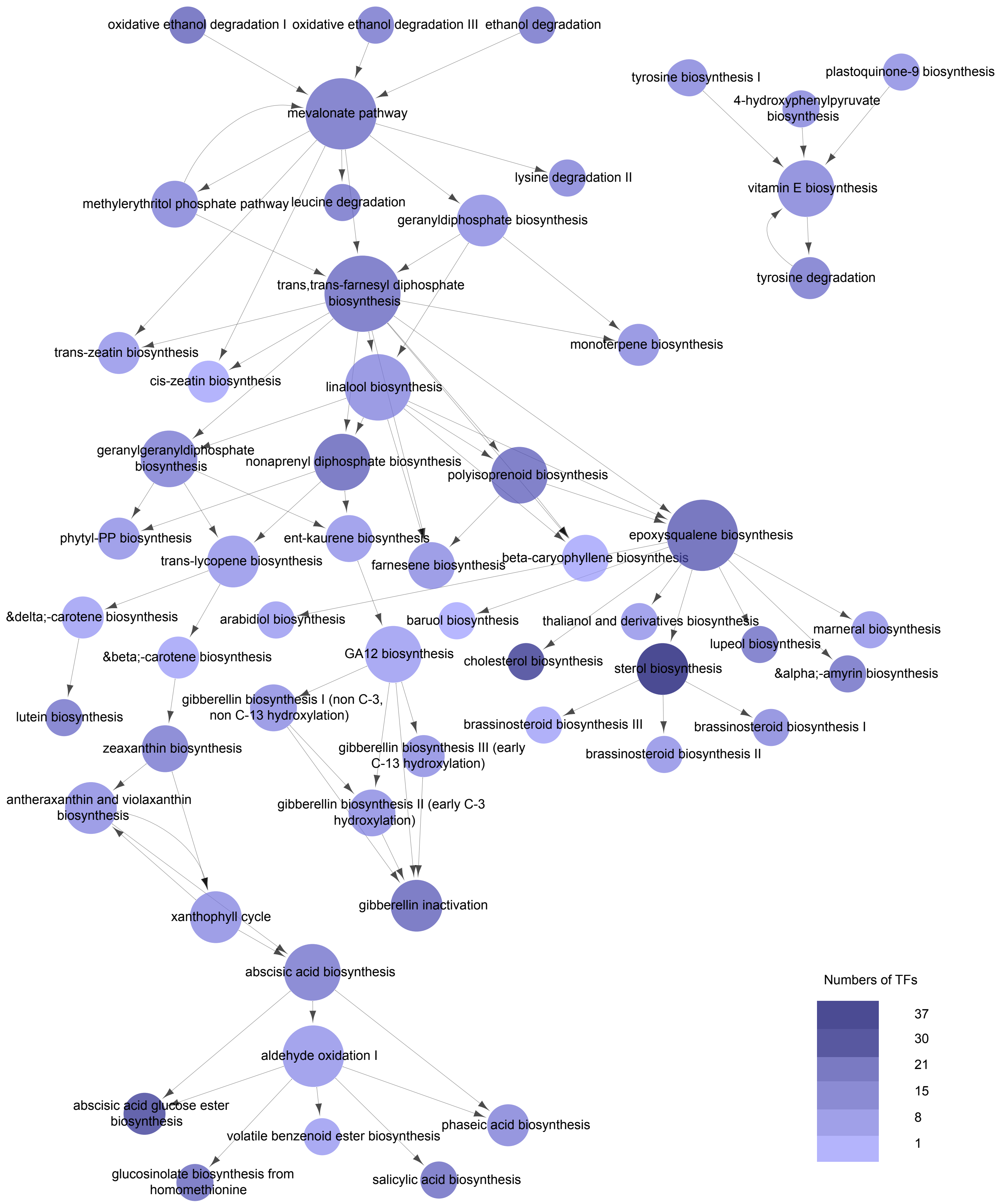

Supplementary Figure S12: Distribution of TFs' numbers in TB metabolic pathway network. (Node size: pathways' degree in metabolic network; Node color: TFs' numbers of pathways)

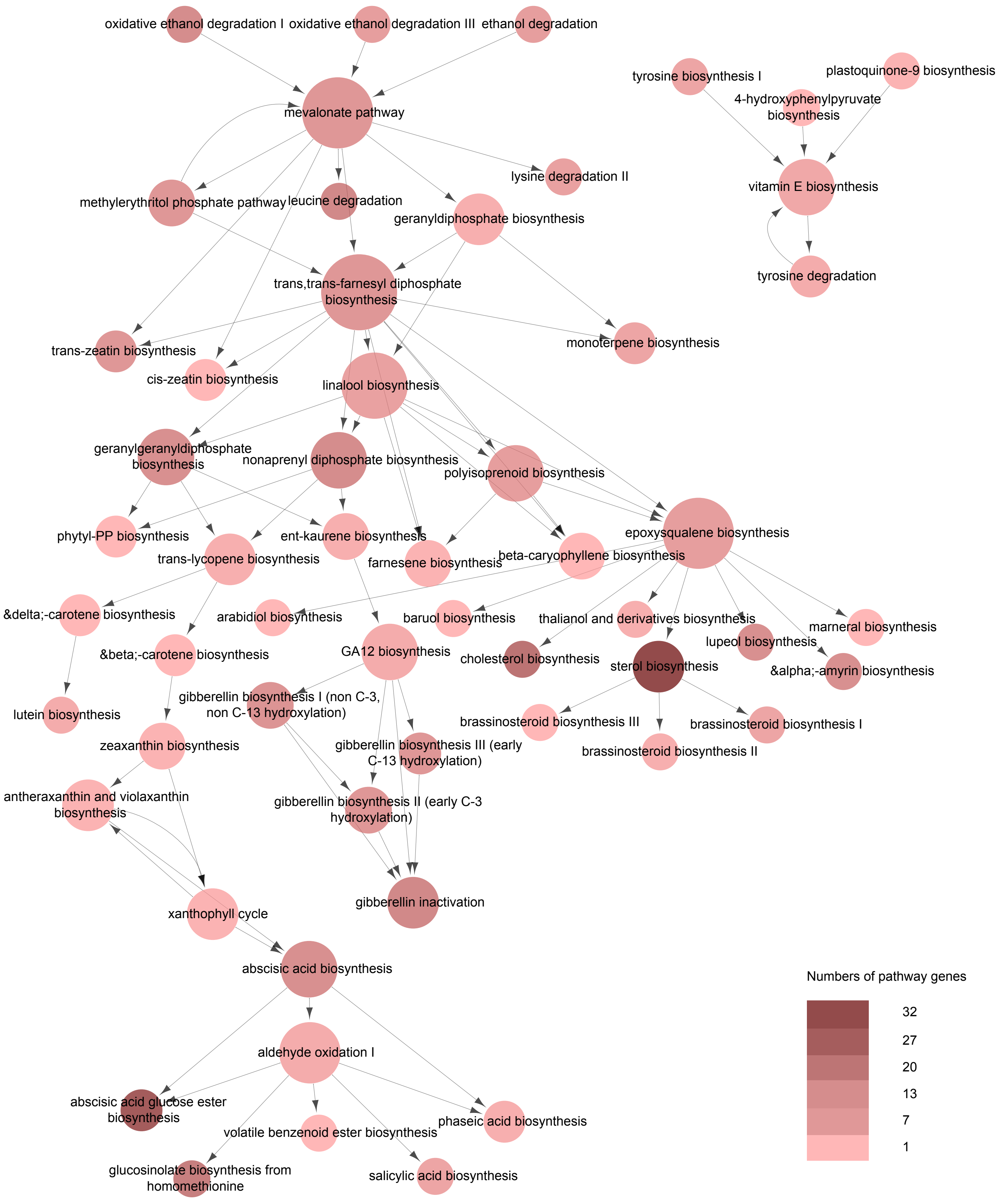

Supplementary Figure S13: Distribution of pathway genes' numbers in TB metabolic pathway network. (Node size: pathways' degree in metabolic network; Node color: pathway genes' numbers of pathways)

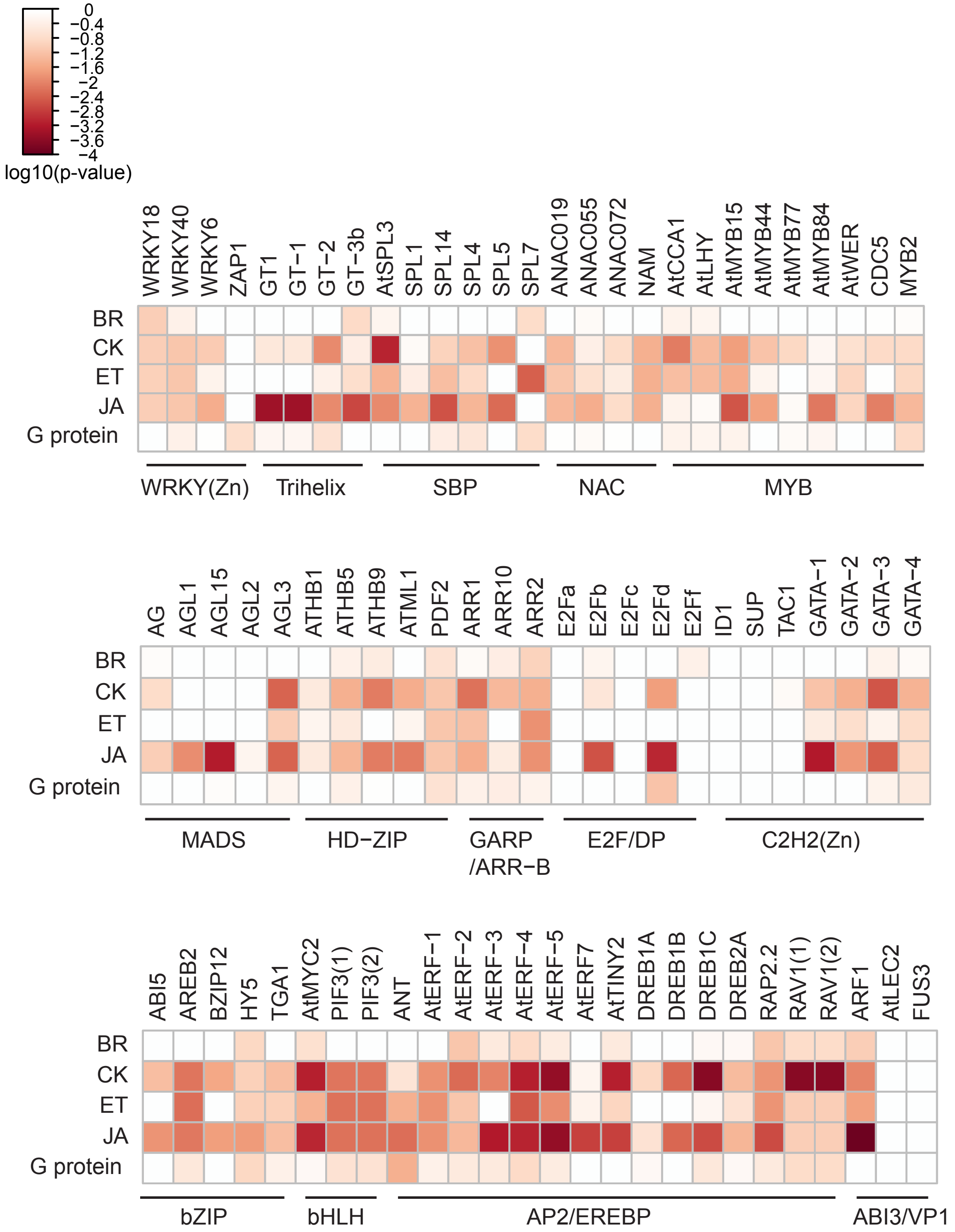

Supplementary Figure S14: Significance of co-expression relatonship between signal pathways and TFs. (BR, Brassinosteroid Signaling Pathway; CK, Cytokinin Signaling Pathway; ET, Ethylene Signaling Pathway; JA, Jasmonate Signaling Pathway; G protein, G Protein Signaling in the Regulation of Arabidopsis Seed Germination.)
